# Screen-Printed Nanohybrid Palladium-Based Electrodes for Fast and Simple Determination of Estradiol in Livestock

**DOI:** 10.1021/acsomega.4c07861

**Published:** 2024-10-31

**Authors:** Claudio Sabbatini Capella Lopes, Francisco Walison Lima Silva, Juliana dos Santos Fernandes, Julia Oliveira Fernandes, João H. A. Ferreira, Felipe Zandonadi Brandão, Ricardo Erthal Santelli, Thiago C. Canevari, Fernando Henrique Cincotto

**Affiliations:** † Departamento de Química Analítica, Instituto de Química, Universidade Federal do Rio de Janeiro, Rio de Janeiro 21941-853, Brazil; ‡ LabNaHm: Multifunctional Hybrid Nanomaterials Laboratory Engineering School, Mackenzie Presbyterian University, São Paulo 01302-907, SP, Brazil; § Faculdade de Veterinária, Universidade Federal Fluminense, Av. Vital Brasil Filho, 64, Niterói, RJ CEP 24230-340, Brazil; ∥ National Institute of Science & Technology of Bioanalytics (INCTBio), Campinas 13083-970, Brazil

## Abstract

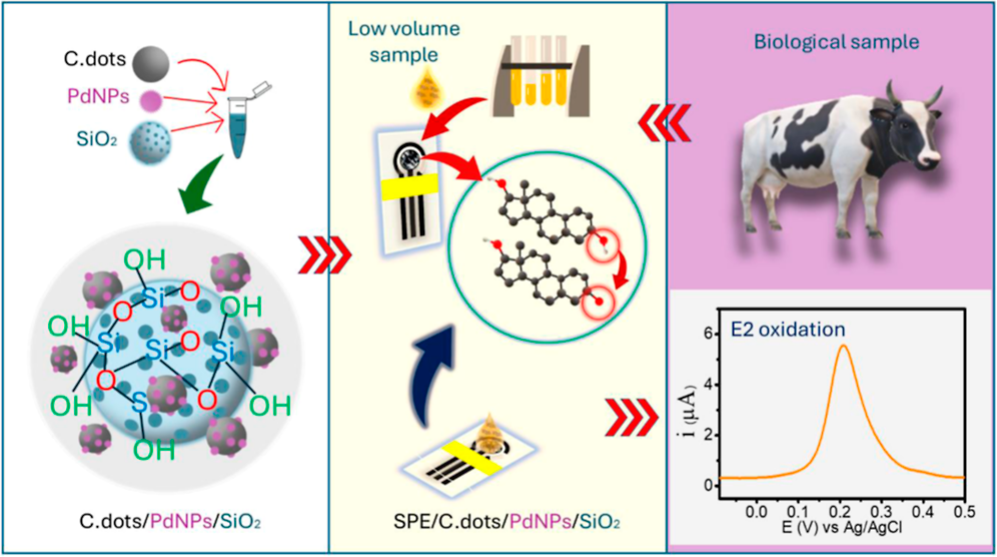

One of the main challenges in animal breeding systems is determining estradiol (E2) in livestock samples as simple and minimally invasive as possible, Thus, a nonenzymatic biosensor screen-printed electrode (SPE) was developed by modifying nanohybrid palladium nanoparticles (PdNPs), and carbon dots anchored on a nanosilica particle (PdNPs/C.dots/SiO_2_), denominated SPE/PdNPs/C.dots/SiO_2_, and successfully tested for the direct detection of estradiol in livestock samples. PdNPs were directly obtained by a one-step synthesis through carbon dot reduction. Hybrid nanomaterials were characterized by atomic force microscopy, high-resolution transmission electron microscopy, and electrochemical impedance. The combination of PdNPs with C.dots resulted in a nonenzymatic biosensor supported on a screen-printed platform with superior electrocatalytic properties regarding the oxidation of E2 when compared to unmodified sensors. Modifications in the working electrode resulted in high sensitivity toward E2 determination within a linear range from 0.005 to 14.0 μmol L^–1^ with a limit of detection of 1.0 nmol L^–1^. The recovery rate of E2 in bovine serum samples and urine samples ranged from 92 to 106%. Interference studies showed that peak current variation (Δ*i*_p_) among all interferents evaluated and E2 did not exceed ±2%. The newly developed sensor stands out not only for its high sensitivity but also for its quick and simple way of production while also being disposable after analysis, providing a simple, sensitive, and practical approach for the determination of reproductive hormones in livestock.

## Introduction

The improvement of zootechnical management of animal farms is part of the concept of precision livestock farming (PLF), which fundamentally is the association of several technical advancements to enhance the health and well-being of farm animals while also ensuring the sustainability and efficiency of farms.^1^ Among the hormones that play an active role in the reproductive cycle of domestic animal species (canine, feline, equine, and bovine), estradiol (E2) is the main hormone regulating the ovulation cycle and behavioral changes in female mammals.^2^ Additionally, within the several veterinarian studies regarding reproductive pathologies in livestock, there is a substantial amount of previous works describing the correlation between plasma concentration of E2 and reproductive disorders.^3,4^

Recently, simple and fast methods are desirable for real-time monitoring of the reproductive cycle of livestock since they can provide valuable information regarding the improvement of zootechnics parameters in farm animals.^5^ The development of disposable electrochemical devices is also useful for detecting biomolecules like glucose, hydrogen peroxide, uric acid, ascorbic acid, dopamine, cholesterol, amino acids, and cancer cells in biochemical systems.^6^ Thus, the development of nonenzymatic biosensors on a screen-printed electrode platform (SPE) with excellent linear range for hormone determination attends to all these requirements with the expend of only a small amount of clinical sample (urine and blood) which is less stressful for livestock compared to traditional assays which require a considerable amount of sample for hormone monitoring,^7^ therefore making electrochemical methods desirable from a welfare animal perspective.

Palladium is a well-known element in catalysis. Their corresponding nanoparticles [palladium nanoparticles (PdNPs)] exhibit excellent catalytic properties, while also favoring, electron transfer and mass transports at the electrode–solution interface.^8^ In addition, PdNPs can be used in C–C coupling reactions,^9^ providing excellent possibilities for developing new electrochemical sensors. However, to improve the electron-transfer process at the surface of SPE, it is possible to modify SPEs, by combining PdNPs with carbon quantum dots (C.dots). These carbon nanocrystals can be obtained by biocompatible routes while also exhibiting good solubility in polar solvents and extensive optic absorption within several visible wavelengths, conferring to these nanocrystals intermediate properties between molecules and semiconductors.^10,11^ Such properties result from the combination of the material type and its particle size, generating a quantum confinement.^12^ Depending on the shape and dimensions, the C.dots may present a certain band gap on the surface of another material with a larger band gap deposited on it. Thus, the electron confined in multiple directions results in a strong quantization of energy levels. This quantization of energy at the point of confinement allows the material to absorb energy with a specific wavelength with energy equivalent to that of the confined electrons.^13^ Furthermore, not only thus C.dots display excellent transfer of electrons but it is also possible to perform simple modifications on their surface to make them suitable for generating different nanostructures, for instance, the combination of C.dots, with PdNPs and silicon-based metal oxides. Silica-based materials are widely used in electrochemical sensors due to their chemical inertness and porosity, facilitating the encapsulation of distinct species and protecting them from the effect of solvents.^14,15^ The presence of several types of silanol groups on the surface of silica-based composites favors mass transport and immobilization of certain nanohybrid particles.^16^ Thus, the objective of the present study is to synthesize nanohybrid materials with PdNPs, C.dots, and silicon dioxide (SiO_2_) nanostructures to modify SPE sensors to obtain a superior sensitivity for measuring the reproductive hormone E2 in livestock samples.

## Materials and Methods

### Chemicals and Reagents

The following reagents were used for the preparation of the nanohybrid materials: absolute ethanol (Neon), K_4_[Fe(CN)_6_] (Dynamic), K_3_[Fe(CN)_6_] (Synth), K_2_HPO_4_, KOH, NaCl, TEOS (Sigma-Aldrich), 28% NH_4_OH (Modern Chemistry), *n*-propanol (Neon), PdCl_2_ (Nuclear), and deionized water (18.2 MΩ, Gehaka OX 50 LX). Chemicals for SPE production: carbon ink, silver chloride ink −99%, obtained from Gwent Electronic Materials (United Kingdom).

### Reagent for Electrochemical Study of Estradiol

Analytical standard for estradiol. Reagents for interference studies: progesterone, glucose, urea, bovine serum albumin, NaCl, CaCO_3_, and KCl (Sigma-Aldrich).

### Apparatus and Procedures

The characterization of the hybrid materials produced was carried out using microscopy images obtained by high-resolution transmission electron microscopy (HR-TEM; JEOL, JEM-2100) and atomic force microscopy (AFM; Molecular Imaging, PicoSPM). The images were taken by the Analytical Center of the Chemistry Institute of the University of São Paulo. The electroanalytical analyses of E2 and electroperformance of the proposed sensor were done in an Autolab PGSTAT101 potentiostat/galvanostat (Metrohm) operating under NOVA 2.1 software (EcoChemie). To construct SPE electrodes, the following materials were employed: several transparency sheets for laser printers and a vinyl adhesive sheet (stationery material), and a cutting printer (Silhouette Cameo 6) was used to produce the SPE mask.

### Synthesis of the Nanohybrid (C.dots/PdNPs/SiO_2_)

The carbon quantum dots (C.dots) were prepared by an electrochemical route, according to a previously reported procedure.^17^ In a 200 mL beaker, 150 mL of *n*-propanol and 12 mL of water were added, and then 1.45 g of KOH was added. The mixture was submitted to an ultrasound bath (37 kHz/50 W) for 20 min to remove the dissolved oxygen in the reaction medium. After this period, two 4 cm^2^ platinum electrodes were introduced into the solution as working and auxiliary electrodes and a calomel-saturated electrode as a reference electrode. The distance between the platinum electrodes was maintained at approximately 1.5 cm. A constant potential of 6.5 V was applied to the working electrode, with a current ceiling of 100 mA, in chronoamperometry mode for 12 h.

For the PdNP synthesis first, palladium chloride (PdCl_2_; 99% Sigma-Aldrich), was first converted to Na_2_[PdCl_4_] using the following procedure: in an Erlenmeyer flask, 2.0 mL of deionized H_2_O, 0.113 mmol of PdCl_2_, and 3.59 mmol of sodium chloride (NaCl, 99%; Sigma-Aldrich) were added. The solution was kept under stirring at room temperature for approximately 30 min until palladium(II) chloride was completely dissolved. Subsequently, 1.0 mL of the Na_2_[PdCl_4_] solution, at a concentration of 56.4 mmol L^–1^, was added to 1.0 mL of H_2_O, and afterward, 3.0 mL of the C.dots suspension was also added and stirred for 1 h at room temperature. During the procedure, it was possible to observe that the color of the solution changed from brown to black in the first 5 min of the reaction, remaining until the end of the stirring time, indicating that there was a chemical transformation in the medium.

The C.dots/PdNPs/SiO_2_ nanostructure was obtained by mixing 1.0 mL of the C.dots/PdNPs solution with 2.5 mL of anhydrous ethanol and 150 μL of tetraethoxysilane (TEOS, 98%; Sigma-Aldrich) and subsequently kept under constant stirring for 30 min at room temperature. Then, 150 μL of 28% NH_4_OH was added, and the solution was kept under stirring again for 20 h. Afterward, the resulting solid material (C.dots/PdNPs/SiO_2_) was separated by centrifugation and washed three times with deionized water. Finally, the material was dried in a vacuum oven at 50 °C. Subsequently, 100 mg of the material was dissolved in 2 mL of ethanol to be used in the modifications of the SPEs.

### Fabrication of SPE/C.dots/PdNPs/SiO_2._

**Scheme 1:**
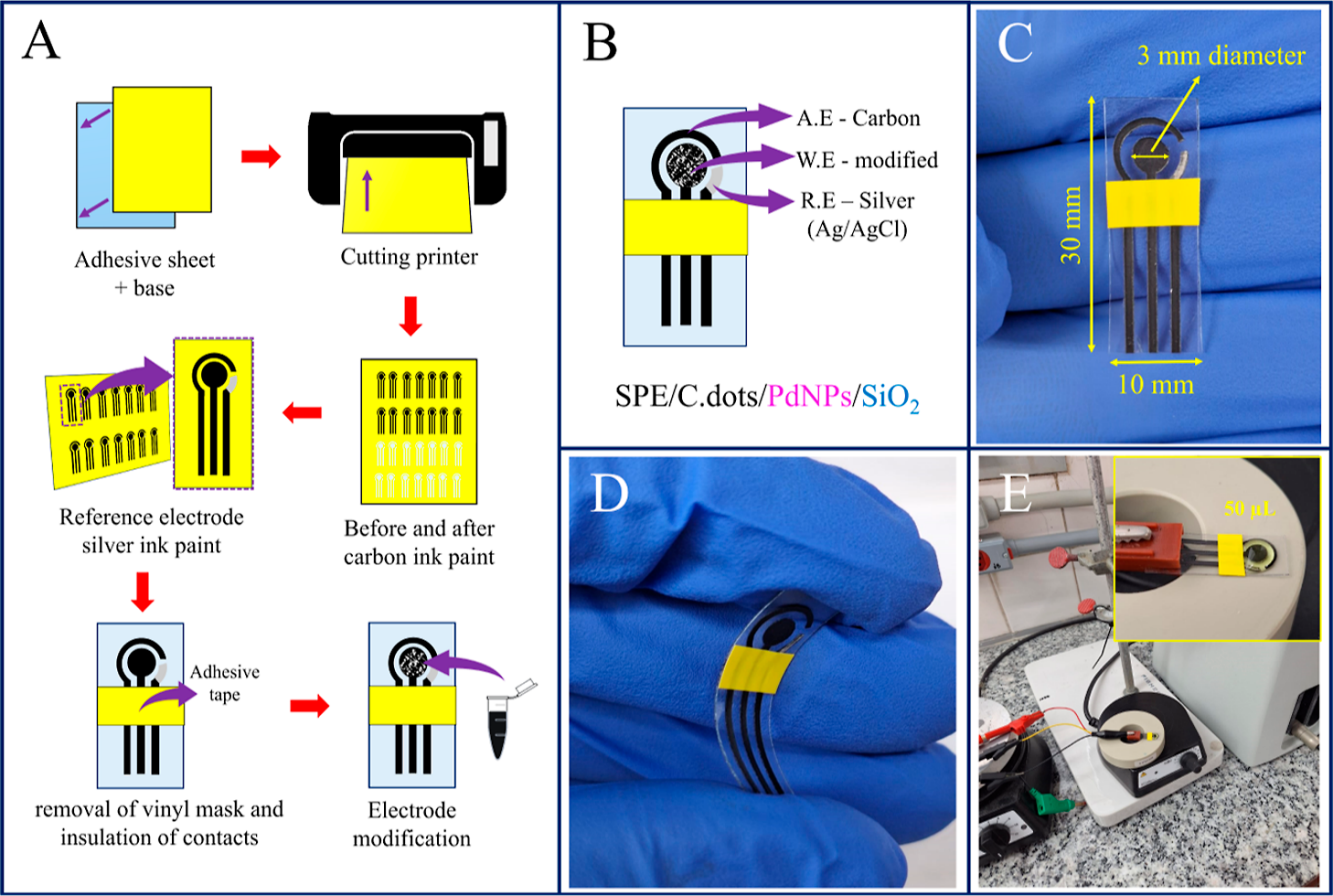
Pictorial View of (A) Fabrication and Modification Procedure of the Screen-Printed Electrodes and (B) Identification of Auxiliary (A.E), Work (W.E), and Reference (R.E) Electrodes. Real View of (C) Screen-Printed Electrode with Dimensions, (D) Flexible Electrode, and (E) Representative Cell with Low Volume of the Sample per Analysis

The fabrication of the SPEs was performed according to the method described by Fernandes *et al.*(18) with modifications. First, a vinyl adhesive sheet was applied over an acetate sheet of the same size and then inserted into a cutting printer to produce the mask (vinyl/acetate) for the disposable electrodes (SPE). Afterward, the vinyl strips previously demarcated by the cutting printer are detached, leaving only the mask for the SPE sensors, which were then coated with carbon paint. Finally, Ag/AgCl paint was applied only over the reference electrode of the SPEs. After the paint had dried, newly produced SPEs were isolated with an adhesive vinyl tape and modified by carefully dropping 10 μL of PdNPs/C.dots/SiO_2_ suspension. The fabrication and modification procedure of the SPEs is illustrated in Scheme 1. After drying the solution at room temperature, the modified SPE/C.dots/PdNPs/SiO_2_ was submitted to electrochemical studies with the analyte E2.

### Construction of the E2 Analytical Curve

The analytical curve for the analyte E2 was obtained from the addition of increasing concentrations of E2 standard solution in Britton–Robinson (BR) buffer 0.2 mol L^–1^ (pH 7.0) in the range of 0.005–14.0 μmol L^–1^ using differential pulse voltammetry (DPV) technique previously optimized. The study was carried out in triplicate, and the anodic peak signal referring to E2 oxidation was correlated to its respective concentration.

### Interference Studies

To determine potential interferents for E2 determination first, a standard solution of E2 (8.0 μmol L^–1^) containing 2.0 mL of BR buffer solution 0.2 mol L^–1^ (pH 7.0) and then several potential interferents for the electrochemical oxidation of E2 in clinical veterinary samples were studied: urea, bovine serum albumin (BSA), glucose, progesterone, K^+^, Na^+^, and Ca^2+^. All potential interferents were individually added to the E2 standard solution at least 10 times the concentration of E2. All experiments were performed in triplicate.

### E2 Determination in Real Samples

The SPE/C.dots/PdNPs/SiO_2_ sensor was used to determine E2 in real serum samples obtained from livestock. The hormone protocol applied to the livestock as well as the blood sample collection and serum extraction was performed at a commercial cattle farm following ARRIVE guidelines. One healthy cow received 2 mg of estradiol benzoate (EB) (Sincrodiol-Ouro Fino; Brazil), and afterward, two blood samples were immediately collected and submitted to centrifugation (1.500 G for 15 min) for serum separation, then stored in 1.5 mL microtubes, and finally stocked at −20 °C. After 24 h, blood samples were once again collected from the same farm animal and submitted to the same serum separation procedure. Then, all resulting serum samples were promptly sent to the lab for electrochemistry experiments. Additionally, recovery of the analyte E2 in serum samples was performed by preparing a standard solution of E2 and then added to serum samples A and B whose final concentrations were 4 and 6 μmol L^–1^, respectively (Table 2). Synthetic urine samples were prepared according to the procedures adapted from Laube *et al.*(19) E2 standard solution was added to samples D and E (Table 2) whose final concentrations were 0.4 and 5.2 μmol L^–1^, respectively.

## Results and Discussion

### Materials Characterization

**Figure 1:**
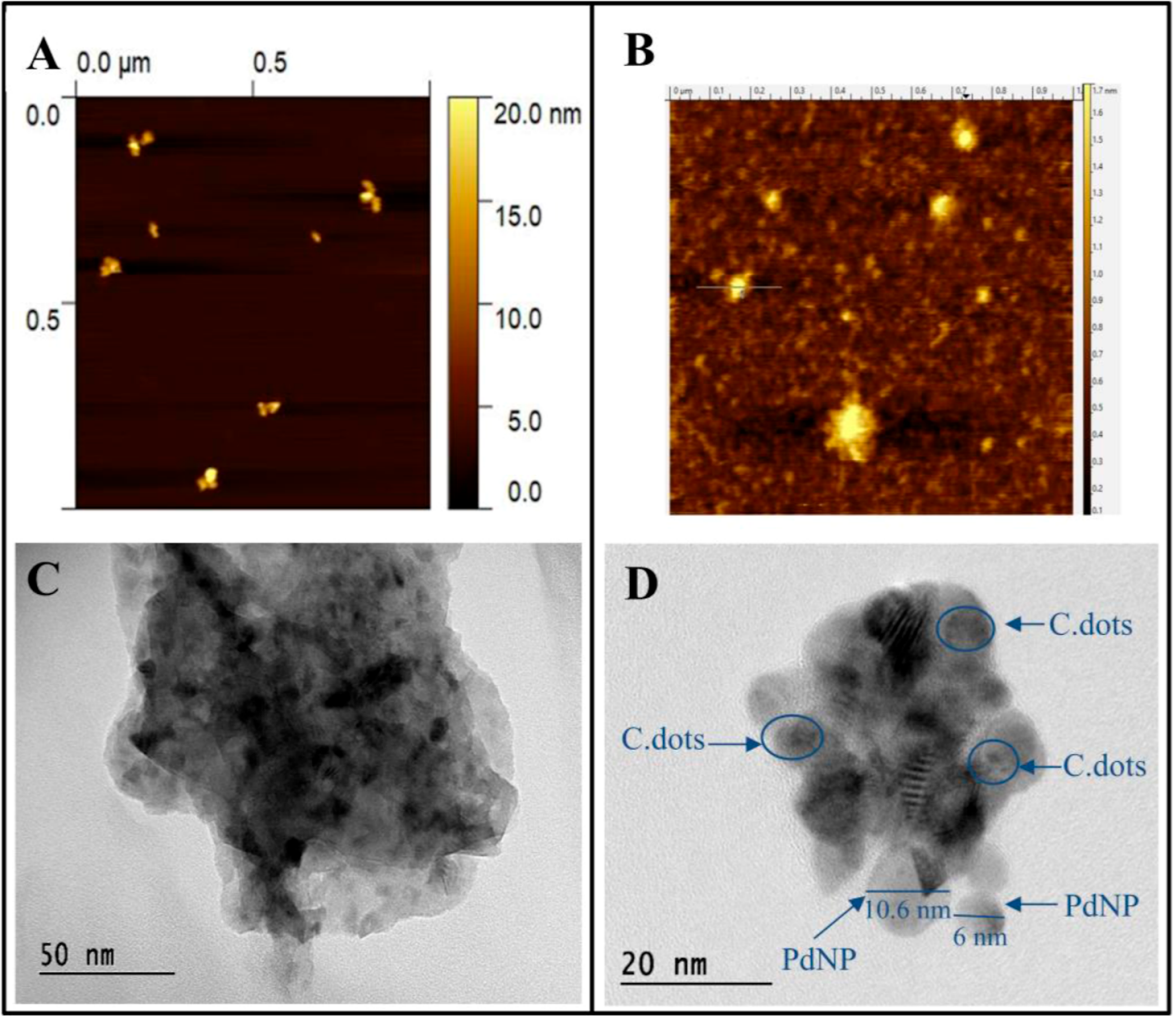
(A) AFM topographic images of C.dots/PdNPs and (B) C.dots/PdNPs/SiO_2_ and (C) HR-TEM micrographic images of C.dots/PdNPs/SiO_2_ and (D) C.dots/PdNPs.

The synthesis of hybrid nanomaterials could be observed after obtaining AFM and HR-TEM images. AFM was used to investigate the surface topography of C.dots/PdNPs (Figure 1A) and C.dots/PdNPs/SiO_2_ (Figure 1B), which revealed a marked difference in the roughness of the surfaces and showed that both C.dots/PdNPs and C.dots/PdNPs/SiO_2_ displayed a flattened shape.

HR-TEM images of C.dots/PdNPs (Figure 1C) and C.dots/PdNPs/SiO_2_ (Figure 1D) showed that the nanomaterials obtained did not have a homogeneous shape. A thin darker layer was observed in the HR-TEM image at the edges of the structure demonstrating the presence of silica in the nanomaterial (Figure 1C). Also, it was possible to confirm the C.dots that covers the entire Pd nanostructure, which have an average diameter of 10.6–6 nm (Figure 1D). These peculiar characteristics of the structures can be explained by the fact that C.dots are both reducing and passivation agents. The XRD patterns of the C.dots/PdNPs/SiO_2_, illustrated in Figure S1, exhibit characteristic peaks for both carbon and PdNPs. The carbon component shows the graphitic phase (002) at 22.4°. The PdNPs are identified by diffraction peaks at 40.5° (012), 47.2° (200), and 68.1° (220), which match the JCPDS standard (No. 05-0681), confirming their presence in the composite (Figure S1).

### Electrochemical Measurements

**Figure 2:**
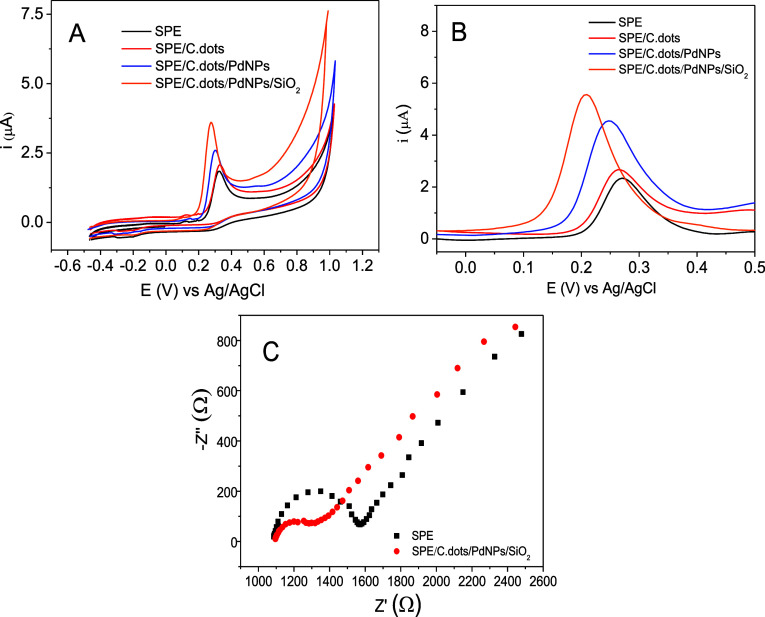
(A) CV and (B) DPV (scan rate of 50.0 mV s^–1^) of the bare SPE sensor and SPE-modified sensors for the determination of E2 (20.0 μmol L^–1^) in BR buffer pH 7.0 (0.2 mol L^–1^). (C) Representative Nyquist plot from EIS measurements of the bare SPE and SPE/C.dots/PdNPs/SiO_2_ sensors at open-circuit potential.

First, cyclic voltammetry (CV) assays were carried out to evaluate the sensitivity of the modified sensors: SPE/C.dots, SPE/C.dots/PdNPs, and SPE/C.dots/PdNPs/SiO_2_ and bare SPE, regarding the capacity to promote a satisfactory electrochemical signal for the oxidation of E2. Additionally, several scans were performed over a wide potential range to characterize the electrochemical behavior of E2 and verify the possibility of more than one oxidation potential. As shown in Figure 2A, the cyclic voltammograms under the conditions already reported demonstrate how hybrid materials can contribute with characteristic current peak signals during the scans. From the conditions studied, the analyte presented only one peak related to the oxidation of the molecule (*E*_E2_ = +0.2 V); therefore, its oxidation was considered irreversible.

Due to its superior sensitivity concerning the oxi-reduction process, the DPV technique was used to carry out a more careful evaluation of the oxidation reaction that occurs at the electrode interface. As can be seen in Figure 2B, the electrochemical properties of the SPE/C.dots/PdNPs/SiO_2_ sensor were studied in BR buffer solution 0.2 mol L^–1^ (pH 7.0). The SPE/C.dots/PdNPs/SiO_2_ sensor presented an outstanding electroperformance regarding the oxidation of the hormone E2 (*E*_E2_ = 0.20 V), when compared to the bare SPEs (*E*_E2_ = 0.27 V), the SPE/C.dots sensor (*E*_E2_ = 0.26 V), and the SPE/C.dots/PdNPs (*E*_E2_ = 0.24 V). The shifting of the electrochemical potential demonstrates that the combination of C.dots/PdNPs/SiO_2_ exhibits superior electrocatalysis properties compared to the other modified stages, which demonstrates an improved efficiency of the electrochemical oxidation process of E2.

The enhanced anodic current implies that there was a positive interaction between the C.dots/PdNPs/SiO_2_ active sites and the analyte E2 on the surface of the electrode. To confirm the increase of the reactive surface of the SPE/C.dots/PdNPs/SiO_2_ sensor, compared to the bare SPE sensor, CV analyses were done using a solution of [Fe(CN)_6_]^3–/4–^ 5 mmol L^–1^ in 0.1 mol L^–1^ KCl at different scan rates (10–125 mV s^–1^) (Figure S2); using the Randles–Sevcik equation: *I*_p_ = (2.69 × 10^5^)*n*^3/2^*AD*^1/2^*cv*^1/2^,^20^ it was possible to estimate the electroactive area of the SPE/C.dots/PdNPs/SiO_2_ sensor at 5.21 × 10^–2^ cm^2^ (Figure S3).

Finally, impedance spectra for the bare SPE sensor and SPE/C.dots/PdNPs/SiO_2_ are shown in Figure 2C. The Nyquist plots of the bare SPE and SPE/C.dots/PdNPs/SiO_2_ indicate a decrease in charge-transfer resistance of 487.5 Ω for 298.6 Ω, respectively. Analyses were realized in 5 mmol L^–1^ [Fe(CN)_6_]^3–/4–^ in KCl 0.1 mol L^–1^ solution. These results demonstrate a significant decrease in the charge-transfer resistance after sensor modification. These data corroborate those obtained in the CV and DPV analyses in the presence of estradiol. The responses obtained in these electrochemical characterization studies demonstrate that the SPE/C.dots/PdNPs/SiO_2_ material presented a synergistic effect that favored the improvement in the electrical transfer at the electrode–solution interface and conductivity.

The synergistic contribution of C.dots/PdNPs/SiO_2_ demonstrated by CV, DPV, and EIS analyses can be explained by the high conductivity of PdNPs, characteristic of metallic nanoparticles, and by the high adhesion of these nanoparticles to C.dots due to the easy interaction with the C–C bonds. In addition, the porosity and silanol groups of SiO_2_ contribute to the dispersion of the material. In general, C.dots/PdNPs/SiO_2_ presents a synergistic contribution due to the combination of characteristics of each material used in the preparation of the nanohybrid.

### Study of pH Influence and Voltammetric Parameters

**Figure 3:**
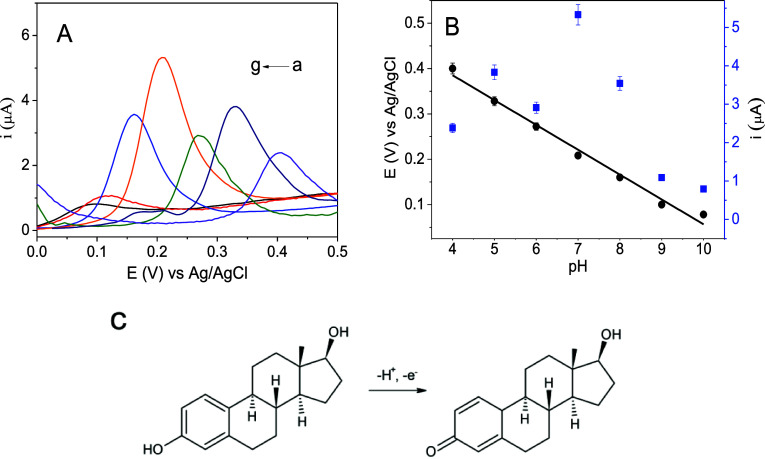
(A) Study of the pH for the oxidation of E2 28 μmol L^–1^ in BR buffer pH (a) 4.0, (b) 5.0, (c) 6.0, (d) 7.0, (e) 8.0, (f) 9.0, and (g) 10.0 (0.2 mol L^–1^) using the SPE/C.dots/PdNPs/SiO_2_ sensor; (B) plot of the DPV peak current (*I*_pa_) and peak potential (*E*_pa_) *versus* pH; and (C) proposed mechanism for the electrooxidation of E2.

To determine the best pH conditions for the oxidation of E2, a series of DPV studies were carried out at the pH range of 4.0–10.0 in BR buffer (0.2 mol L^–1^) containing 28.0 μmol L^–1^ E2 to observe the best conditions for a satisfactory analytical signal in the determination of E2. All analyses were performed in triplicate. According to Figure 3A, it was possible to observe a superior anodic peak corresponding to BR buffer pH 7.0 in addition to a higher resolution and peak height, while also providing an analytical signal at a lower potential when compared to BR buffer acid solutions (pH 6 at 4). In addition, according to the plot of *I*_pa_*vs* pH (Figure 3B—blue points), the peak current has an optimum value at pH 7.0 and steadily decreases for higher pH, while a less evident decrease is observed for lower pHs. Thus, pH 7.0 was chosen for subsequent experiments using the SPE/C.dots/PdNPs/SiO_2_ sensor.

The optimization of DPV parameters is crucial to increment the sensitivity of the proposed sensor in the present work. Potential increment, modulation amplitude, and time modulation (Figures S4–S6) were carefully investigated for the SPE/C.dots/PdNPs/SiO_2_ sensor (BR buffer pH 7.0; 0.2 mol L^–1^ containing 28 μmol L^–1^). Based on the present results, a step potential of 5 mV was chosen due to its higher peak potential; it was also observed that a higher step potential led to lower anodic peak currents regarding the oxidation of E2, while a lower step potential resulted in a loss of peak definition, loss of baseline, and an increase in time analysis. Afterward, the step potential was fixed at 5 mV, and modulation amplitude was tested from 10 to 100 mV, the results showed that an increase in peak current could be correlated with the increase in the peak amplitude, and thus, the pulse amplitude of 100 mV was chosen for the following experiments. After fixing both step potential and modulation amplitude, the contribution of time modulation toward peak definition of oxidation of E2 was evaluated in the range of 20–100 ms, and a period of 50 ms was chosen due to its superior peak current and baseline definition.

### Performance of Analytical Response

**Figure 4:**
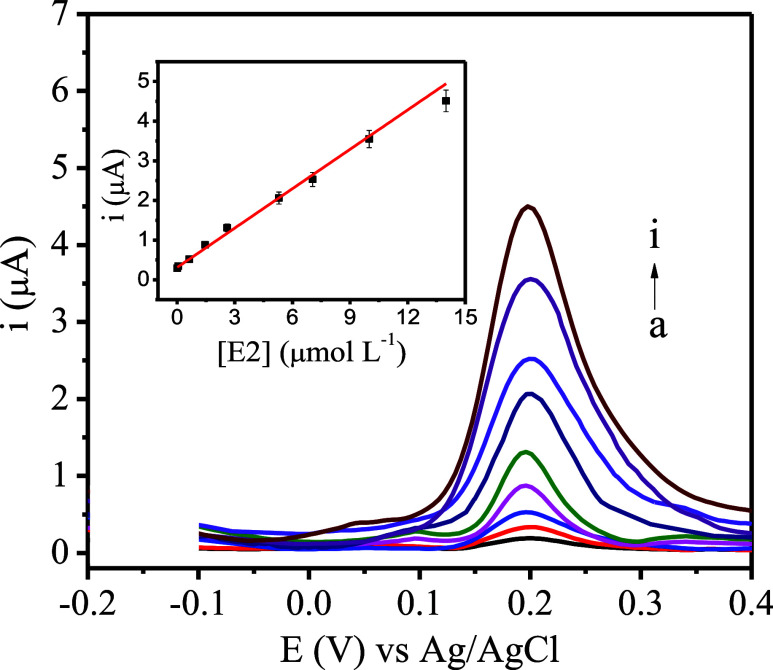
Analytical curve for SPE/C.dots/PdNPs/SiO_2_ for the determination of E2 in BR buffer, pH 7.0, with the following concentrations for E2: (a) 0.005; (b) 0.06; (c) 0.63; (d) 1.45; (e) 2.60; (f) 5.30; (g) 7.05; (h) 10.0; and (i) 14.0 μmol L^–1^ (inset: calibration curve).

The SPE/C.dots/PdNPs/SiO_2_ sensor showed high sensitivity to E2 determination in the linear range from 0.005 to 14.0 μmol L^–1^ with a limit of detection (LOD) of 1.0 nmol L^–1^, calculated by the equation LOD = 3σ/*b*,^23^ where σ is the standard deviation obtained for the blank (*n* = 10) and *b* is the slope of the analytical curve. There was a proportional increase in the anodic current (μA) as a function of the increase in the concentration (μmol L^–1^) of E2. As shown in Figure 4, the analytical curve corresponds to the quantification of E2, which resulted in the equation *i*_pa_ (μA) = 0.330 ± 0.013 [E2 (μmol L^–1^)] + 0.311 ± 0.01, *R*^2^ = 0.990.

Table 1 presents a series of electrochemical devices which have been employed for direct measurement of E2. It is possible to observe that the SPE/C.dots/PdNPs/SiO_2_ displayed a superior LOD for the determination of E2 when compared with most electrochemical sensors. Recently, Musa *et al.*,^24^ reached a detection limit of 0.041 μmol L^–1^ for estradiol through the technique of CV using graphene screen-printed electrodes; also, Galvão *et al.*(25) were able to establish a detection limit of 0.004 μmol L^–1^ for estradiol, using a glassy carbon electrode (GCE) with a nanocomposite consisting of α-Fe_2_O_3_ nanoparticles supported on carbon nanotubes (CNTs), with the SWV technique. In the present study, the modified sensor exhibited a superior LOD compared to these previous works. It is also worth mentioning that not only did the present sensor stand out for its high sensitivity but also for its quick and simple way of production while also being disposable after analysis, which avoids possible poisoning of the electrode, which can lead to a reduction of the sensitivity and reproducibility of the analysis, which usually occurs in nondisposable electrodes such as those based on glassy carbon due to constant cleaning.

**Table 1:** Comparison between the SPE/C.dots/PdNPs/SiO_2_ Sensor and Other Electrochemical Devices for E2 Determination

electrode	technique	linear range (μmol L^–1^)	LOD (μmol L^–1^)	ref
aGHSPE	CV	0.83–4.98	0.0041	(24)
bα-Fe_2_O_3_–CNT/GCE	SWV	0.005–0.1	0.004	(25)
cG-C_3_N_4_/AuNPs/ITO	CV	25–600	6.5	(31)
dCFME’s	FSCV	0.01–100	0.03	(32)
eFe_3_O_4_–NC/GCE	DPV	0.01–20.0	0.0049	(33)
fCPE/CeO_2_ NPs	SWV	0.01–0.1	0.0013	(34)
gNPEDMA	CV	0.1–0.8	0.686	(35)
hSPE/C.dots/PdNPs/SiO_2_	DPV	0.005–14.0	0.001	this work

a Graphene screen-printed electrodes.

b GCE with a nanocomposite consisting of α-Fe_2_O_3_ nanoparticles supported on CNTs.

c Graphitic carbon nitride (g-C_3_N_4_) assembled on a gold nanoparticle/indium tin oxide film electrode.

d Carbon fiber microelectrodes.

e GCE modified with Fe_3_O_4_-doped nanoporous carbon.

f Graphite sensor modified by cerium oxide nanoparticles.

g *N*-Phenylethylene diamine methacrylamide.

h SWV: Square wave voltammetry. CV: Cyclic voltammetry. DPV: Differential pulse voltammetry. FSCV: Fast scan cyclic voltammetry.

Although blood concentrations of estradiol in cows and heifers are from 0.004 nmol L^–1^ (1 pg/mL) to 0.030 nmol L^–1^ (8 pg/mL) during the luteal phase of the estrous cycle,^26^ there are several issues regarding estradiol in biochemical paths where screen-printed electrochemical sensors could play a role by providing rapid and concise information regarding the concentration of estradiol. Recently, there has been reports of the influence of nanomolar concentrations (0.01–10 nmol L^–1^) of estradiol in mitogen-activated protein kinase (MAPK) pathway in pituitary cells and thus anticipating the luteal phase of bovine’s estrus cycle, thus leading to cows or heifers more promptly to enter the ovulation period which is beneficial for farmland breeding by improving herd profitability and reduction of the overall interbreeding interval and reproductive program cost.^27^ Also, in a clinical controlled experiment, Sajiki *et al.*(28) reported that cows and heifers in different estrus cycle stages and infected with bovine leukemia virus, an enzootic retrovirus that causes enzootic bovine leukosis, received exogenous doses of estradiol and afterward had their peripheral blood mononuclear cells collected. The concentrations of estradiol ranged from 2.36 to 7.09 nmol L^–1^, the authors reported that high concentration of estradiol had a positive effect toward the induction of prostaglandin PGE_2_ to suppress immune response pathway Th1 of all the animals, during the pregnancy and parturition stage, leading to the progression of BLV. Therefore, based on the LOD presented by SPE/C.dots/PdNPs/SiO_2_, it is possible to apply the modified sensor in metabolism studies providing a quick and practical response regarding the role of estradiol in such clinical conditions.

Also, now a days, there has been public awareness concerning estrogens levels in the environment, which has attracted many scientific reports worldwide, addressing aspects to the source of estrogen contaminations. Thus, the use of miniaturized screen-printed electrodes with exceptional LOD is a practical approach to determine sampling sites in farmlands or in the environment. He *et al.*(29) reported that over 90% of the estrogens detected in the environment are derived from animal farming. More recently, Tang *et al.*(30) studied the environmental risks associated with the discharge of livestock containing natural estrogens; the authors reported that the daily urinary excretion rates of estradiol by dairy cattle was 28.7 ng/mL (104.6 nmol L^–1^), while in pregnant beef cattle urine, the concentration of estradiol was around 44.8 ng/mL (163.3 nmol L^–1^). Therefore, the use of modified screen-printed electrochemical devices with excellent LOD could be useful for sampling in livestock environments.

### Reproducibility and Stability

In this study, the reproducibility and stability of the SPE/C.dots/PdNPs/SiO_2_ sensor were evaluated by DPV technique in the presence of 6.0 μmol L^–1^ of E2 in BR buffer 0.1 mol L^–1^ (pH 7). Reproducibility analyses were performed with five different electrodes, showing an RSD (relative standard deviation) of 2.85%, indicating excellent consistency in measurements among the electrodes (Figure S7A). The stability of the SPE/C.dots/PdNPs/SiO_2_ sensor was evaluated at intervals of 1, 5, 10, 15, and 20 days, with results demonstrating an RSD of 5.41% (Figure S7B). These results highlight the feasibility of using the proposed sensor in the E2 determination.

### Interference Studies

**Figure 5:**
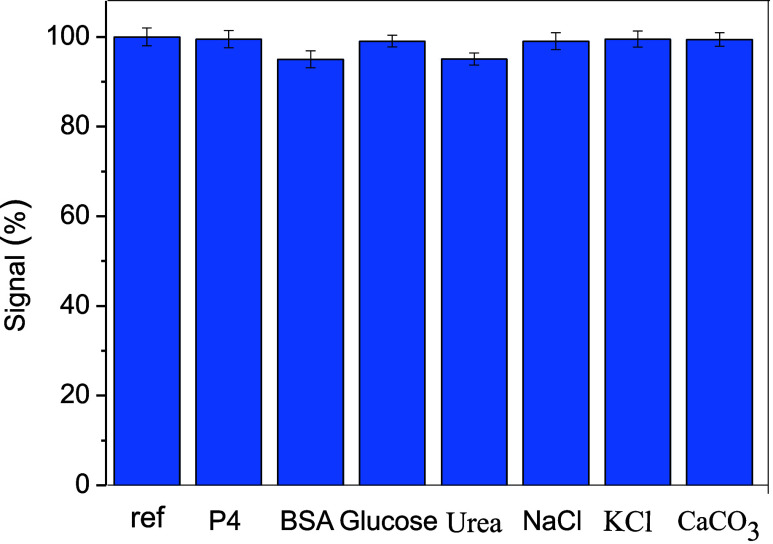
Study of potential interferences in the determination of E2 in the presence of progesterone (P4); BSA; glucose; urea; Na^+^; K^+^; and Ca^2+^.

To evaluate possible interference from matrix components concerning the selectivity of the SPE/C.dots/PdNPs/SiO_2_ sensor toward the determination of E2, several possible interferents were studied, such as progesterone, BSA, glucose, urea, NaCl, KCl, and CaCO_3_. All interferents were added individually with E2 at a concentration at least 10 times higher than the concentration of the analyte; all experiments were performed in triplicate. The result demonstrates that the variation in peak current (Δ*i*_p_) between each interferent and E2 did not exceed ±2% as observed in Figure 5. Also, the proposed sensor exhibited a satisfactory selectivity toward the oxidation of E2 in the presence of each interferent and thus demonstrated both organic and inorganic compounds which are commonly present in livestock clinical samples and did not promote significant alterations in the anodic peak current of E2.

### E2 Determination in Real Samples

The direct determination of E2 in real cow serum samples and synthetic urine using the SPE/C.dots/PdNPs/SiO_2_ sensor was evaluated by the DPV technique previously optimized. The recovery rate of E2 in cow serum samples was 92 and 93%, respectively, while the recovery rate in synthetic urine was 97 and 106% (Table 2). Ultimately, given that the determination of the reproductive hormone E2 did not suffer any significant matrix effect, these results demonstrate that the SPE/C.dots/PdNPs/SiO_2_ sensor can be applied in field analysis, thus providing valuable information regarding the estrus cycle of livestock within breeding seasons. According to Morgante *et al.*,^36^ the average pH blood of domestic ruminants such as bovines and caprines ranges from 7.3 to 7.44 ± 0.005; therefore, the conditions achieved in the present study for the oxidation of E2 favors the application of the electrochemical method in blood samples of livestock. Therefore, the present results demonstrate the possibility to determine livestock hormones in a fast straightforward way while also maintaining a good state of animal welfare.

**Table 2:** Results for Recovery of E2 in Bovine Serum and Synthetic Urine Samples

	added (μmol L^–1^)	found (μmol L^–1^)	recovery (%)
bovine serum (C)		3.38	
bovine serum + spike 1 (A)	4.0	7.08	93.0 ± 1.4^a^
bovine serum + spike 2 (B)	6.0	8.88	92.0 ± 1.2^a^
synthetic urine (F)			
synthetic urine + spike 1 (D)	0.4	0.42	106.0 ± 1.0
synthetic urine + spike 2 (E)	5.2	5.0	97.0 ± 1.5

a E2 was found in the bovine serum real sample; thus, the recoveries for the bovine serum-spiked real samples were calculated based on the following equation: recovery (%) = [(found (μmol L^–1^) – 3.38 μmol L^–1^)/added (μmol L^–1^)] × 100%.

## Conclusions

In the present study, a new printed and disposable electrochemical device was proposed for the determination of estradiol: SPE/C.dots/PdNPs/SiO_2_. The novel nanohybrid material, based on PdNPs, carbon quantum dots, and silicon oxide, showed good attachment properties toward the SPE surface, and electrochemistry synergism, which contributed to the direct oxidation of E2. The sensor showed high selectivity in the linear range from 0.005 to 14.0 μmol L^–1^, with a limit of detection of 1.0 nmol L^–1^. The proposed sensor exhibited a good selectivity toward the determination of E2 in the presence of commonly organic and inorganic interferents present in livestock samples. The modified sensor presented an excellent recovery rate when submitted to real bovine serum samples and synthetic urine samples. Therefore, the combination of a disposable, simple, sensitive, and low-cost electrochemical sensor could be applied to determine low levels of estradiol in biological samples, which opens the possibility to use this modified sensor to quantify estradiol in biochemical studies involving livestock, providing a quick and practical response regarding the role of estradiol in such processes. As a highlight, the screen-printed electrode presented shows compatibility for connection with portable potentiostat; thus, the use of smartphones can be combined with the developed method through direct connection or by using Bluetooth technology. Also considering the significant amount of estrogen discharge into the environment by livestock, the present work demonstrates the possibility for future applications of screen-printed electrodes in the field of hormone monitoring in farmland sludges.

Figure 3B (black points) presents the linear correlation between *E*_pa_ and pH, indicating a trend regarding the anodic peaks to potentials closer to zero, as the pH solution increased, resulting in a decreased energy, is required for the oxidation of E2. In addition, the equation obtained by the linear correlation between the oxidation potential and pH (*E* (V) = −0.055 pH + 0.604) expresses an angular coefficient of −0.055; this value is very similar to the theoretical value of the Nernst equation *E*_p_ = (0.0592*m*/*n*)pH + *b*,^21^ where it is expressed as the characterization ratio of one electron and one proton (1:1) for the E2 oxidation reaction on the SPE/C.dots/PdNPs/SiO_2_ sensor. This result is also corroborated by a previous work developed by Cincotto *et al.*(11) where the authors report that the electro-oxidation of the reproductive hormone 17-β estradiol is governed by the transfer of equal numbers of electrons and protons. Additionally, regarding the electrochemical behavior of the analyte, the oxidation of the hydroxyl group in the aromatic ring of the E2 molecule generates its corresponding ketone derivative.^22^ This irreversible behavior is attributed to the transfer of electrons from the estradiol oxidation reaction to the surface of the electrode. The proposed mechanism is presented in Figure 3C.

## Data Availability

The data underlying this study are available in the published article and its Supporting Information.

## Supporting Information Available

XRD analysis for the C.dots/PdNPS material; study of the influence of the scan rate in the presence of the [Fe(CN)_6_]^3–/4–^ 5 mmol L^–1^ in 0.1 mol L^–1^ KCl with the C.dot/PdNPs/SiO_2_ sensor; studies of the optimization of the DPV technique parameters (step potential, modulation amplitude, and modulation time); and DPV voltammograms of the reproducibility and stability (PDF)

## Notes

The present study was approved by the Brazilian Ethical Committee for Animal Use (CEUA: Comitê de Ética no Uso de Animais; file no. 6485210423). Available at: https://www.icmbio.gov.br/cepta/pesquisa/ceua-comissao-de-etica-no-uso-de-animais.html. CEUA follows the ARRIVE guidelines. All experiments were performed in accordance with the U.K. Animals (Scientific Procedures) Act, 1986, and associated guidelines, EU Directive 2010/63/EU for animal experiments.
