# Supplementary material for: Screen-Printed Nanohybrid Palladium-Based Electrodes for Fast and Simple Determination of Estradiol in Livestock
Source: ACS Omega. 2024 Oct 31;9(48):47729–38. doi: 10.1021/acsomega.4c07861 (PMC11618398; doi:10.1021/acsomega.4c07861)
Supplement: Supplementary file 1 — ao4c07861_si_001.pdf [file ao4c07861_si_001.pdf]

Suplementary material

Screen-printed nanohybrid palladium-based electrodes for fast and simple determination of estradiol in livestock

Claudio Sabbatini Capella Lopes[a], Francisco Walison Lima Silva[a], Juliana dos Santos Fernandes[a], Julia Oliveira Fernandes[a], João H. A. Ferreira[b], Felipe Zandonadi Brandão[c], Ricardo Erthal Santeli[a,d], Thiago C. Canevari[b], Fernando Henrique Cincotto[a,d]

[a]*Departamento de Química Analítica, Instituto de Química, Universidade Federal do Rio de Janeiro, Rio de Janeiro, Brazil*

[b]*LabNaHm: Multifunctional Hybrid Nanomaterials Laboratory Engineering School, Mackenzie Presbyterian University, 01302-907, São Paulo, SP, Brazil*

[c]*Faculdade de Veterinária, Universidade Federal Fluminense, Av. Vital Brasil Filho, 64, Niterói, RJ CEP 24230-340, Brazil*

[d]*National Institute of Science & Technology of Bioanalytics (INCTBio), Campinas, Brazil*

*e-mail of the corresponding author: fernandocincotto@gmail.com

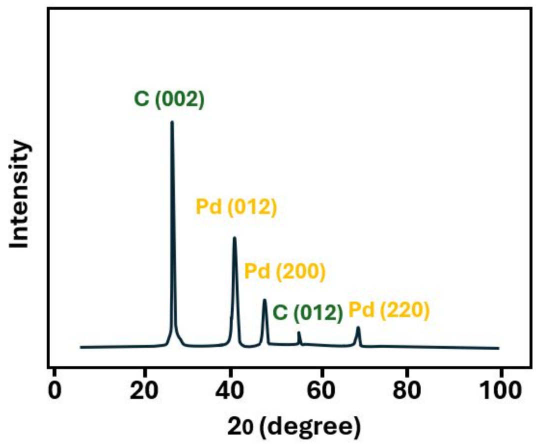

Figure S1 – XRD analysis for the C.dots/PdNPS material according to JCPDS standard (No. 05-0681)

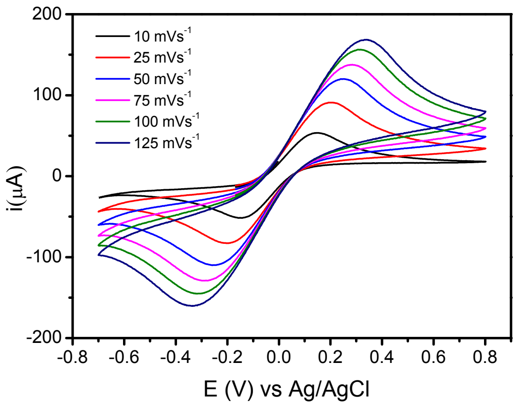

Figure S2 - CV analyses using a solution of $[Fe(CN)_6]^{3-/4-}$ 5mmol L^{-1} in 0.1 mol L^{-1} KCl at different scan rates (10 - 125 mV s^{-1}) for the PdNPs/C.dots/SiO_2 sensor.

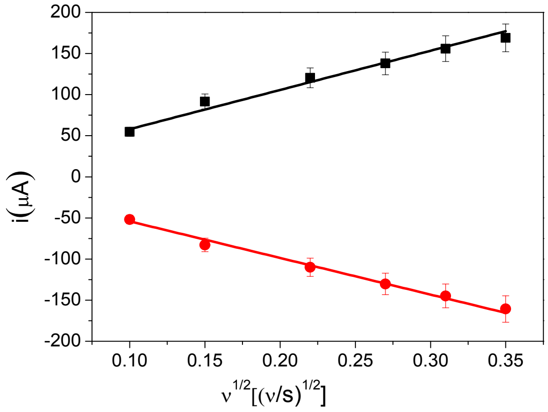

Figure S3 - Linear fitting [E(V) vs iµA)], for application of the Randles-Sevcik equation as toestimate the electroactive area of the SPE/PdNPs/C.dots/SiO_2 sensor, compared to the bare SPE sensor.

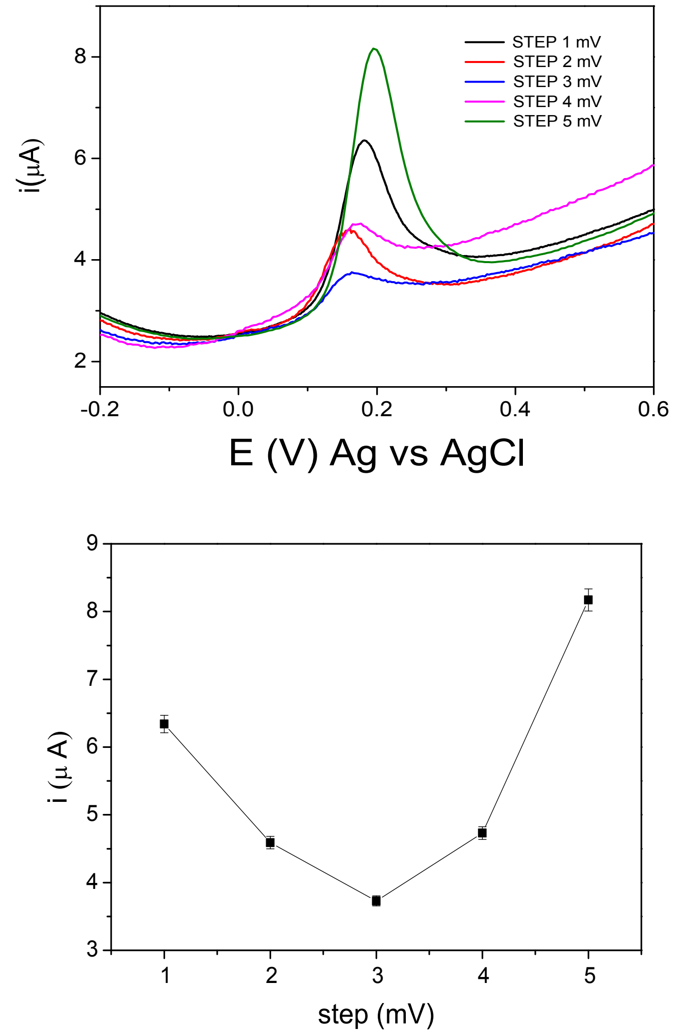

Figure S4 - Potential increment modulation for the SPE/PdNPs/C.dots/SiO_2 sensor.

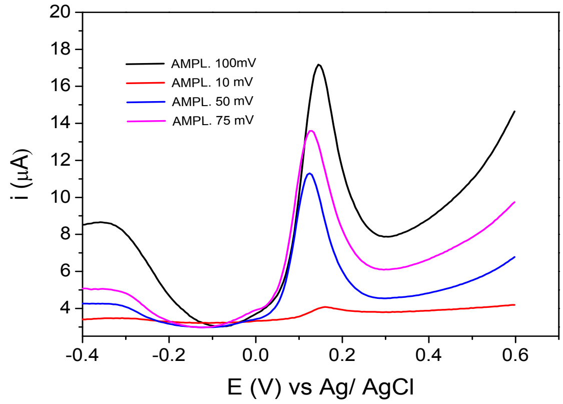

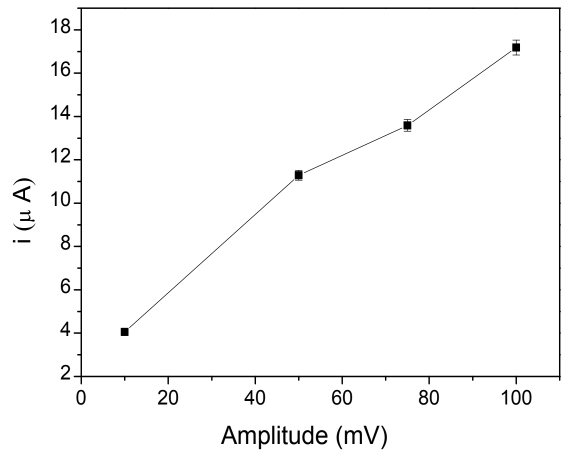

Figure S5 - Modulation amplitude for the SPE/PdNPs/C.dots/SiO_2, sensor

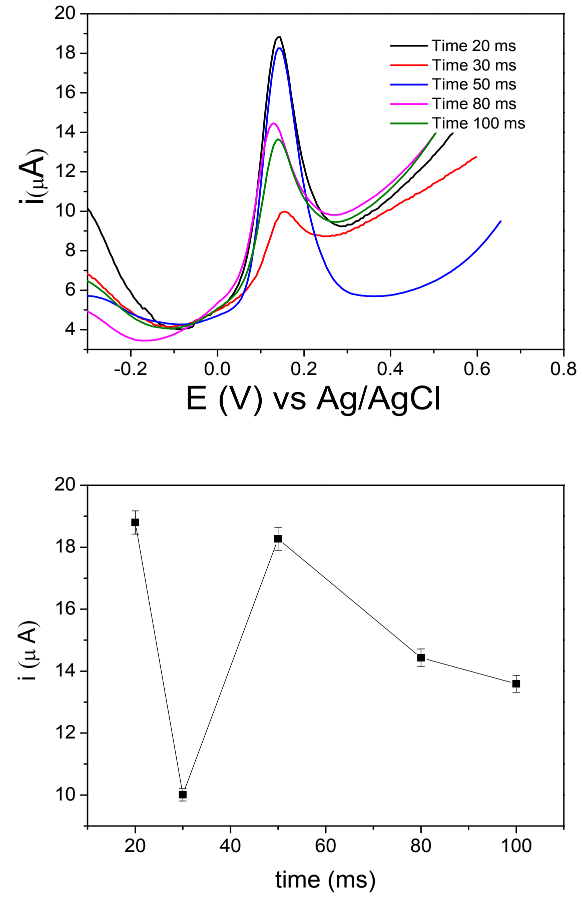

Figure S6 - Time modulation for the SPE/PdNPs/C.dots/SiO_2 sensor

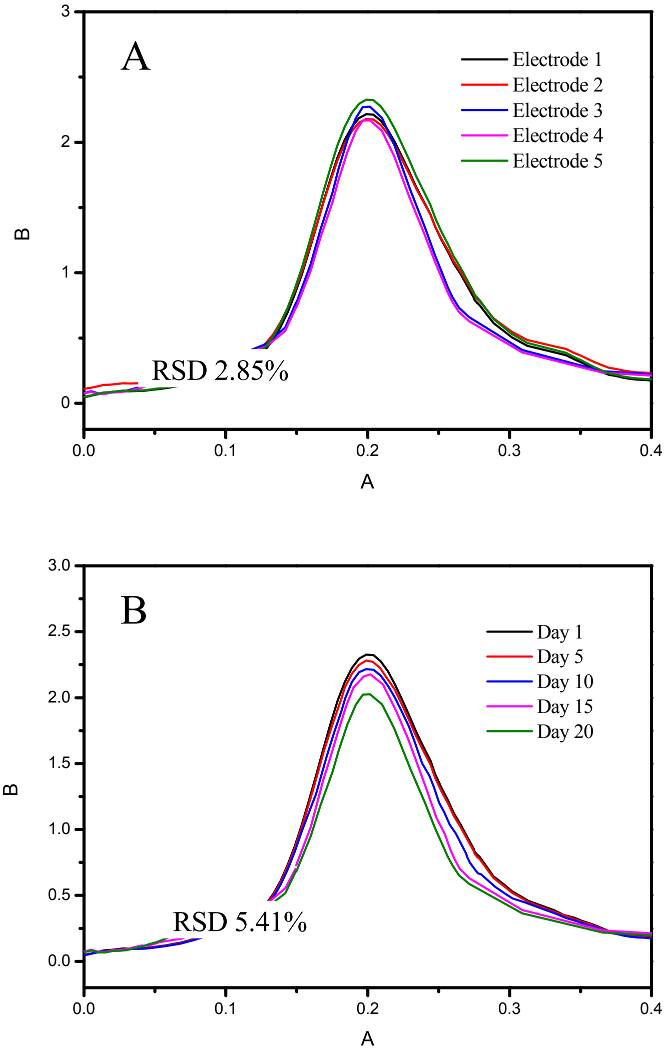

Figure S7 – DPV voltammograms of the: (A) reproducibility and (B) stability
